# Cryptogenic Multifocal Ulcerating Stenosing Enteritis (CMUSE) in a Patient with Down Syndrome: A Case Report

**DOI:** 10.3390/reports9030231

**Published:** 2026-07-20

**Authors:** Akash Bharatbhai Patel, David Zula, Karan Varshney, Daryl Thompson, Vladamir Bolshinsky

**Affiliations:** 1 Peninsula University Hospital, Bayside Health, Frankston, VIC 3199, Australia; abpatel@phcn.vic.gov.au (A.B.P.);; 2 School of Public Health and Preventive Medicine, Monash University, Melbourne, VIC 3004, Australia; 3 Dorevitch Laboratories, Bayside Health, Frankston, VIC 3199, Australia

**Keywords:** CMUSE, cryptogenic jejunitis, small bowel strictures, Down syndrome, enterocutaneous fistula, case report

## Abstract

**Background and Clinical Significance**: Cryptogenic multifocal ulcerating stenosing enteritis (CMUSE) is a rare idiopathic disorder of the small bowel which remains diagnostically challenging because it can closely mimic Crohn’s disease, celiac disease, and non-steroidal anti-inflammatory drug (NSAID)-induced enteropathy; **Case Presentation**: We report a 44-year-old man with Down syndrome, Hirschsprung’s disease, celiac disease, and multiple prior abdominal operations who developed recurrent small-bowel strictures of uncertain cause. Initial management involved endoscopic assessment and jejunal dilatation, but this became neither technically feasible nor durable as the disease progressed. He therefore underwent exploratory laparotomy with small-bowel resection to relieve obstruction and to obtain adequate tissue for diagnosis. On balance, the presence of multifocal ulceration, recurrent mucosa-predominant strictures, and non-transmural jejunitis supported a diagnosis of CMUSE; **Conclusions**: This case highlights the rarity and diagnostic difficulty of CMUSE, which may closely resemble Crohn’s disease in patients with recurrent small-bowel strictures and obstructive symptoms. Early and ongoing MDT coordination (surgery, gastroenterology, radiology, dietetics, and infectious diseases) supports anatomy definition, complication control, and coherent long-term management focused on function and quality of life.

## 1. Introduction and Clinical Significance

Cryptogenic multifocal ulcerating stenosing enteritis (CMUSE) is an uncommon idiopathic disease characterized by multiple fibrous strictures and superficial ulcerations of the small intestine, first described in 1964 [1,2,3,4,5,6]. CMUSE typically presents with recurrent subacute small-bowel obstruction, chronic abdominal pain, and malnutrition [7,8,9,10].

Although CMUSE can involve the small bowel at more than one level, published series describe involvement of the jejunum, ileum, or both, with many reports emphasizing jejunal and proximal ileal predominance and relative sparing of the colon—an anatomic pattern that can help distinguish it from Crohn’s disease, which more commonly involves the terminal ileum (often with colonic disease) [7,11,12,13]. Unlike Crohn’s disease, CMUSE lacks transmural inflammation, granulomas, or extraintestinal manifestations but its pathogenesis remains unclear, though proposed mechanisms include immune dysregulation, impaired mucosal repair, and genetic predisposition [14,15].

The condition is notoriously difficult to diagnose, being frequently misclassified as Crohn’s disease or celiac disease. Management is challenging because responses to corticosteroids and biologics are variable: corticosteroids may induce partial or short-term improvement in some patients, but relapse, steroid dependence, and inconsistent biologic efficacy have been reported. Patients often undergo repeated surgical interventions that may result in the cumulative risk of short bowel syndrome.

We report a case of CMUSE in a patient with Down syndrome and Hirschsprung disease, highlighting diagnostic dilemmas, therapeutic difficulties, and implications for surgical practice.

## 2. Case Presentation

A 44-year-old man with Down syndrome presented with nausea, vomiting, and worsening abdominal pain, four weeks after an exploratory laparotomy with small-bowel resection performed for multifocal strictures. His past medical history included Hirschsprung’s disease treated in the neonatal period with left hemicolectomy, with subsequent stoma formation and later reversal; celiac disease; dural venous sinus thrombosis on apixaban; and prior Helicobacter pylori gastritis (treated). He had undergone multiple previous laparotomies, including a gastrojejunostomy performed for gastric outlet obstruction and prior small-bowel procedures (including strictureplasty) undertaken for recurrent obstructive symptoms due to stricturing small-bowel disease. A chronological summary of events is shown in Table 1.

The gastroenterology team referred him to the colorectal team after an Inflammatory Bowel Disease Multidisciplinary Meeting review to revisit the working diagnosis (Crohn’s vs. CMUSE/other) and to obtain surgical assessment of the stricturing burden with consideration of targeted, bowel-preserving intervention (e.g., stricturoplasty). Colorectal input was sought for definitive tissue diagnosis via planned full-thickness small-bowel/mesenteric biopsy, preceded by optimization with an elective TPN admission (~2 weeks) and a prednisolone wean to enhance biopsy yield and wound healing. The team was also asked to evaluate and guide management of the new colocutaneous fistula in the context of prior colorectal surgery, and to advise on sequencing/timing of any operative strategies in a high-risk, malnourished patient with dense adhesions and a prior gastrojejunostomy. Ancillary requests included staging strictures with a barium swallow/meal and arranging a ^68^Ga-DOTATATE PET scan given the elevated gastrin and chromogranin A to exclude a gastrin-producing Neuroendocrine Tumor.

### 2.1. Examination

On examination, he was 38.2–38.9 degree Celsius febrile and tachycardic at 100–130, with a distended abdomen and tenderness around his midline laparotomy wound. Laboratory investigations demonstrated leukopenia (<4 × 10^9^/L), anemia (65–100 g/L), elevated C-reactive protein (75–270 mg/L), and hypoalbuminemia (11–22 g/L). Initial imaging revealed multifocal small-bowel strictures with gastric distension and small intra-abdominal collections, consistent with ongoing inflammation and partial obstruction. Notably, several weeks after his most recent small-bowel surgery, he developed a colocutaneous fistula with effluent draining via the prior midline laparotomy wound. Although this initially appeared unexpected in the absence of recognized colonic involvement at the index operation, subsequent clinical assessment considered the fistula to be more plausibly related to the patient’s previous colorectal surgery for Hirschsprung’s disease most likely originating from the prior coloanal/rectal anastomosis rather than representing a new intraoperative colonic injury. This delayed and atypical postoperative complication significantly complicated his course and created substantial diagnostic and management challenges.

### 2.2. Investigations

Over a two-year period and multiple admissions, the patient underwent extensive investigations to clarify the etiology of recurrent small-bowel strictures and to exclude mimicking conditions, including Crohn’s disease, celiac disease, infectious enteropathies, and malignancy. Following the initial gastrojejunostomy (December 2023), duodenal biopsies demonstrated chronic active severe enteritis with ulceration, without intraepithelial lymphocytosis, granulomas, dysplasia, or malignancy—features not supportive of Crohn’s disease or celiac disease. With persistent symptoms, repeat endoscopy and biopsies (August 2024) showed extensive mucosal ulceration with granulation tissue formation and fibrosis, accompanied by neutrophilic inflammation; importantly, there were no granulomas, viral inclusions, or parasites. Special stains (PAS, Grocott, Ziehl–Neelsen) were negative for fungal or mycobacterial infection, and immunohistochemistry excluded CMV and HSV.

**Figure 1:**
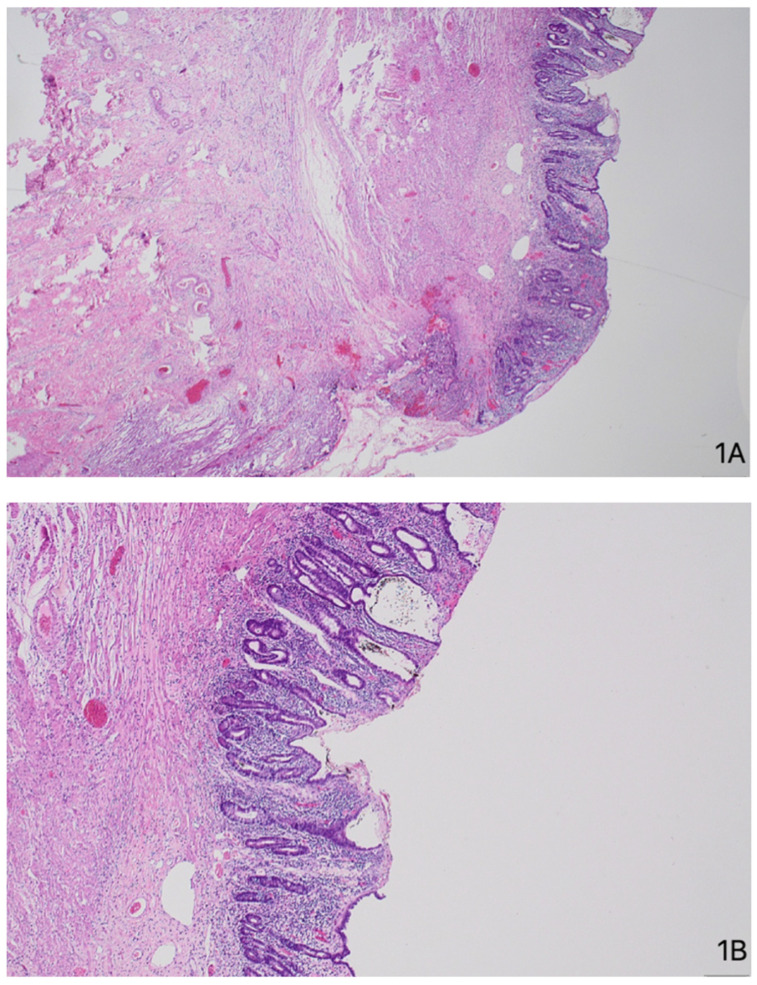
Jejunum, H&E at (**A**) 20× magnification and (**B**) 100× magnification showing ulceration, granulation tissue, and villous architectural distortion. Inflammation is predominantly superficial rather than transmural and no granulomata are identified (as would be expected in Crohn’s disease).

**Figure 2:**
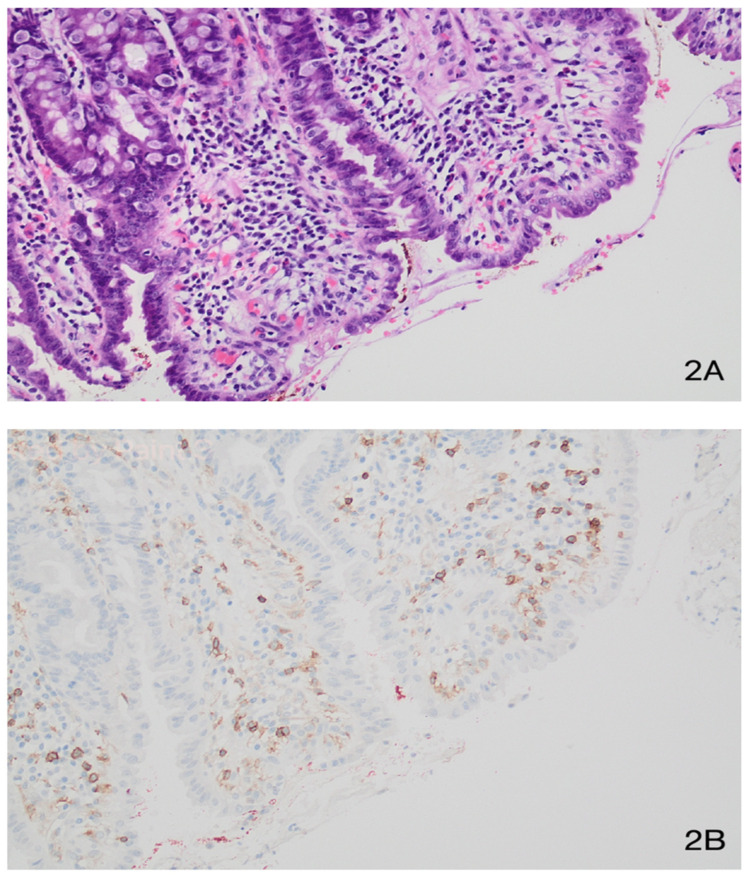
Jejunum, at 200× (**A**) H&E and (**B**) CD4 immunohistochemistry showing an absence of intraepithelial lymphocytosis (as would be expected in celiac disease).

During the same admission, duodenal biopsy demonstrated focal mild duodenitis with preserved villous architecture, excluding celiac disease and Whipple’s disease. In July 2025, following another episode of small-bowel obstruction, resection specimens confirmed chronic active jejunitis with patchy mucosal ulceration confined to the mucosa and submucosa, with crypt distortion, pseudo-pyloric metaplasia, cryptitis, and crypt abscesses; there was no transmural inflammation, granulomas, or intraepithelial lymphocytosis, and all eleven lymph nodes sampled were reactive only. Subsequently, in September 2025, tissue obtained from wound debridement demonstrated hyper granulation with inflammatory changes, without evidence of malignancy. Endoscopic assessments were concordant with histology: gastroscopy in January 2024 (post-gastrojejunostomy) identified four short jejunal strictures approximately 40 cm distal to the anastomosis, which were dilated to 15 mm, and gastroscopy in August 2025 demonstrated a patent anastomosis but a tight narrowing 20–30 cm beyond that was impassable to the scope; nasogastric decompression and nasojejunal tube insertion were performed under direct vision. Views of the jejunum with findings inconsistent with Crohn’s disease are shown in Figure 1, and findings inconsistent with celiac disease are shown in Figure 2.

Serial CT abdomen/pelvis studies (2024–2025) demonstrated multifocal small-bowel strictures with proximal dilatation and matted loops, consistent with recurrent obstructive episodes and supporting a diffuse stricturing enteropathy rather than a single fixed anastomotic lesion. Imaging did not identify a discrete perforation. In August 2025, CT again showed marked gastric distension with fluid-filled dilated small-bowel loops, in keeping with partial obstruction. Following subsequent wound breakdown (September 2025), CT demonstrated hypodense material tracking along the laparotomy wound with extensive adjacent inflammatory change and matted bowel loops, raising suspicion for an evolving enteric cutaneous fistula.

Laboratory evaluation throughout his course demonstrated chronic anemia (Hb 67–114 g/L), reactive thrombocytosis (platelets 400–600 × 10^9^/L), and intermittent neutrophilia, consistent with recurrent inflammation and infection. C-reactive protein remained persistently elevated (up to 265 mg/L) during septic and obstructive episodes. Renal function remained stable, with only transient electrolyte disturbances. Liver biochemistry showed mild, fluctuating transaminitis and hypoalbuminemia (<30 g/L), reflective of chronic inflammation and malnutrition. Blood cultures grew *Staphylococcus lugdunensis* on one occasion, which was later regarded as a contaminant; all other cultures were negative. Repeated celiac serology was negative, and no viral or mycobacterial pathogens were detected histologically.

Collectively, the findings across serial endoscopic, radiological, and histopathological evaluations demonstrated chronic ulcerating jejunitis with multifocal, non-transmural strictures and no evidence of Crohn’s-like, infectious, or celiac pathology—thereby supporting a final diagnosis of cryptogenic multifocal ulcerating stenosing enteritis (CMUSE), reached as a diagnosis of exclusion.

### 2.3. Management

The patient’s management required a complex, multimodal approach involving repeated surgery, escalation of medical therapy, nutritional optimization, and close multidisciplinary coordination. He had previously undergone several laparotomies, including open adhesiolysis and gastrojejunostomy for gastric outlet obstruction in December 2023, followed by an exploratory laparotomy with adhesiolysis, strictureplasty, and jejunal resection for multifocal small-bowel strictures in July 2025. His early postoperative recovery was complicated by wound dehiscence and breakdown, necessitating eventual formal wound debridement with washout in September 2025.

Persistent vomiting and intolerance to oral intake prompted repeat endoscopic assessment, which confirmed a patent gastrojejunostomy but demonstrated a tight narrowing 20–30 cm beyond the anastomosis that was impassable to the scope. Nasogastric decompression was therefore instituted, and a nasojejunal feeding tube was inserted to deliver enteral nutrition distal to the obstruction. In the weeks that followed, the patient developed a colocutaneous fistula through the midline wound—an unusual and delayed complication given the absence of any identified colonic injury during prior operations. Given his poor nutritional status and high surgical risk, operative re-exploration was deferred in favor of conservative management with fistula bagging, drainage of collections where appropriate, and ongoing wound care.

From a medical standpoint, the patient was treated with systemic corticosteroids (prednisolone and budesonide) to control mucosal inflammation, although these were temporarily withheld postoperatively to facilitate wound healing. Infliximab therapy was trialed but demonstrated limited efficacy, in keeping with published experience in CMUSE, and alternative biologics were avoided due to his history of dural venous sinus thrombosis. Recurrent infective episodes were managed with broad-spectrum antibiotics (initially piperacillin–tazobactam, later de-escalated to oral trimethoprim–sulfamethoxazole following *Staphylococcus lugdunensis* bacteremia) under the guidance of the infectious diseases team.

Given recurrent vomiting, partial obstruction, and protein–calorie malnutrition, total parenteral nutrition (TPN) was initiated under dietetic supervision. Enteral feeding via the nasojejunal route was trialed when feasible but frequently limited by distal strictures. Long-term outpatient TPN via a peripherally inserted central catheter (PICC) was considered to maintain nutritional status.

The fistula was managed nonoperatively with bowel rest (nil per os), meticulous fluid and electrolyte replacement, and local wound care with intent to suppress hypergranulation. Sepsis screening remained negative, and there were no features documented to suggest distal obstruction. Nutritional optimization was prioritized given severe hypoalbuminemia (albumin 18–21 g/L). Enteral intake was withheld initially, with gradual reintroduction once the effluent decreased and abdominal findings remained quiescent. The fistula exhibited high output characteristics early in the course but down-trended steadily with conservative measures, ultimately closing spontaneously without endoscopic or operative intervention.

On 14 October 2025, the patient underwent an emergency procedure for an infected prior laparotomy wound with excessive granulation tissue, consisting of excision of granulation tissue, wound washout, and drainage of an adjacent abscess. A loculated abscess left of the laparotomy wound was identified, drained, and sent for pathology with ongoing intravenous antibiotic management. On 28 October 2025, interventional radiology revised the existing 14 Fr fistula drain due to its superficial position; with anesthesia-provided sedation, the old drain was removed over a wire, with contrast injection demonstrating a complex pelvic fistula pattern without a dominant cavity or collection, with a new 14 Fr drain being advanced deeper. On 14 November 2025, to delineate anatomy for suspected colocutaneous fistula (with IR drain in situ), colonoscopy/ileoscopy under general anesthesia demonstrated approximately 60 cm of colon and 20 cm of ileum examined; an ileal polyp was removed by cold snare, and random ileal and colonic biopsies were obtained. A left-sided diverticulum at ~55 cm from the anal verge was biopsied with the apparent defect being closed and an old anastomosis at ~15 cm (consistent with prior Hirschsprung surgery) was biopsied via a visible defect. Concomitant wound assessment showed inflammatory tissue only, with no cellulitis and no requirement for debridement.

Regular multidisciplinary team (MDT) meetings involving surgery, gastroenterology, dietetics, infectious diseases, and radiology guided ongoing management. The consensus was to avoid further radical resections, given the high recurrence rate of CMUSE and the risk of short bowel syndrome. Long-term management focused on symptom control, nutritional support, infection prevention, and judicious use of immunosuppression to preserve quality of life.

### 2.4. Outcome/Follow-Up

Despite intensive surgical and medical management, the patient experienced recurrent strictures, wound dehiscence, and intra-abdominal sepsis. He remains under multidisciplinary care involving gastroenterology, infectious diseases, dietetics, and surgery, with plans for long-term nutritional support and close surveillance for recurrent complications.

## 3. Discussion

CMUSE is an uncommon entity with relatively few cases reported, and the central clinical problem is its close phenotypic overlap with Crohn’s disease [1,2,3,4,5,6,7,8,9,10]. Getting this distinction right matters, because an incorrect Crohn’s label can commit a patient to prolonged immunosuppression without addressing the true disease biology [15,16]. In this case, Crohn’s disease was considered but was not supported by the overall clinicopathological pattern: strictures were multifocal and recurrent, but histology showed superficial ulcerating jejunitis with mucosal and submucosal involvement rather than transmural inflammation, granulomas, fissuring ulcers, or Crohn’s-like lymphoid aggregates. Celiac disease was also unlikely on the available work-up, with negative celiac serology and no intraepithelial lymphocytosis on histology.

The pathogenesis of CMUSE remains incompletely defined, which contributes to diagnostic uncertainty and therapeutic variability [15,16,17]. Current hypotheses converge on impaired mucosal integrity and defective healing responses as plausible drivers of the recurrent superficial ulceration and stenosis. In parallel, emerging genetic data have implicated variants in SLCO2A1, which may disrupt prostaglandin transport and metabolism, offering a biologically coherent mechanism linking dysregulated prostaglandin signaling to abnormal mucosal repair and recurrent stricturing in at least a subset of patients [13,15,17,18,19,20,21]. However, it must be noted that the authors were not able to conduct genetic testing for this, as such testing was not available; this serves as a notable limitation of our work. On this note, the principal limitation of our work is the nature in which CMUSE is a diagnosis of exclusion, and—though many of the most common possible differential diagnoses were ruled out in our work, it was not possible to rule out all other possibilities. It is possible that other, rare chronic enteropathies may have contributed to the presentation, such as those associated with the SLCO2A1 gene—an example being chronic enteropathy associated with the SLCO2A1 gene (CEAS), which has numerous investigation findings (such as histological, radiological and endoscopic findings) and clinical characteristics that overlap with CMUSE. Therefore, in our case, there are limits regarding the diagnostic certainty of CMUSE.

The coexistence of Down syndrome in this case should also be interpreted cautiously. To our knowledge, a consistent association between Down syndrome and CMUSE has not been established. However, Down syndrome is associated with immune dysregulation, including interferon-driven immune activation, and with increased susceptibility to autoimmune gastrointestinal disease such as celiac disease [22]. A recent case series described adults with Down syndrome and celiac disease who developed enteritis and small-intestinal strictures, although CMUSE was excluded in those cases [23]. Accordingly, we present Down syndrome here as relevant clinical context that contributed to diagnostic complexity, rather than as a proven pathogenic driver of CMUSE.

Histopathology is central to this distinction. CMUSE classically produces multiple shallow ulcers and short strictures, with inflammation concentrated in the mucosa and submucosa. In contrast, Crohn’s disease more typically demonstrates transmural chronic inflammation, granulomas in a subset of cases, fissuring ulceration, creeping fat, fistulising behavior arising from transmural disease, and extraintestinal features. The absence of granulomas, transmural inflammation, viral inclusions, parasites, dysplasia, malignancy, and active celiac-type intraepithelial lymphocytosis in this case supported CMUSE/CMUSE-like enteropathy over Crohn’s disease, celiac disease, infection, or malignancy.

Treatment evidence for CMUSE remains limited and is largely derived from case reports and small series. Corticosteroids may induce partial or short-term improvement in some patients, but steroid dependence and relapse during tapering are common [11,12,13,14,15,16]. Immunomodulators and biologics, including anti-TNF therapy, have shown inconsistent benefit, and no standard disease-modifying regimen has been established. Other reported strategies are individualized and include nutritional support, endoscopic dilatation where technically feasible, bowel-sparing stricturoplasty, and limited resection for fixed obstructing segments. In our patient, corticosteroids and budesonide were used, infliximab produced limited clinical benefit, and nutritional optimization became central because recurrent obstruction and sepsis compounded malnutrition.

Long-term outcomes in CMUSE are shaped by recurrence, malnutrition, anemia, repeated obstructive episodes, and the cumulative consequences of intervention. Surgery can relieve obstruction and provide tissue for diagnosis, but it is not curative; recurrent strictures and repeat operations increase the risk of short bowel syndrome. For this reason, the long-term objective is not eradication of disease but preservation of intestinal length, prevention and early treatment of sepsis, nutritional maintenance, judicious immunosuppression, and sustained multidisciplinary follow-up. This case illustrates those challenges, with recurrent stricturing, wound morbidity, fistula formation, and ongoing need for coordinated surgical and gastroenterology care.

Comparison with reported CMUSE cases and series highlights the same diagnostic pitfalls and follow-up challenges seen in this patient. Prior reports describe recurrent abdominal pain, anemia, hypoalbuminemia, protein-losing enteropathy, small-bowel ulceration, multifocal strictures, and repeated obstructive episodes, often after Crohn’s disease, celiac disease, infection, malignancy, and non-steroidal anti-inflammatory drug enteropathy have been considered and excluded [1,2,11,16]. Several cases have required endoscopic dilatation, segmental resection, or bowel-sparing stricturoplasty for symptomatic strictures, but relapse and recurrent obstruction remain common [11,15,16]. Our case parallels these reports in its recurrent stricturing phenotype and diagnostic reliance on clinicopathological exclusion, but is additionally complicated by Down syndrome, Hirschsprung’s disease with prior colorectal surgery, malnutrition, postoperative sepsis, and fistula formation. These features reinforce the importance of careful diagnosis, avoidance of unnecessary long-term Crohn’s-directed therapy when pathology is not supportive, and structured longitudinal follow-up focused on nutrition, recurrence surveillance, and preservation of bowel length.

## 4. Conclusions

CMUSE is a rare but severe cause of recurrent small-bowel obstruction, often misdiagnosed as Crohn’s disease or celiac disease. CMUSE should be considered in patients with recurrent multifocal small-bowel strictures and ulceration, particularly when the clinical course and histopathology are not consistent with Crohn’s disease. This case illustrates the difficulty in diagnosis, limited success of medical therapies, and the high morbidity associated with repeated surgical interventions. Early recognition, careful surgical planning, and multidisciplinary management are vital to improving patient outcomes.

## Figures and Tables

**Table 1:** Chronological summary of major clinical events, investigations, interventions and outcomes.

Date/Period	Clinical Event, Investigation or Intervention	Outcome/Interpretation
Neonatal period	Hirschsprung’s disease treated with left hemicolectomy, stoma formation and later reversal.	Established the complex colorectal surgical background relevant to later fistula assessment.
Prior to 2023	Multiple previous laparotomies for recurrent obstructive symptoms, including prior small-bowel procedures/strictureplasty.	Recurrent stricturing small-bowel disease with persistent diagnostic uncertainty.
December 2023	Open adhesiolysis and gastrojejunostomy for gastric outlet obstruction. Duodenal biopsies showed chronic active severe enteritis with ulceration, without granulomas, dysplasia, malignancy or intraepithelial lymphocytosis.	Findings were not supportive of Crohn’s disease or active celiac disease.
August 2024	Repeat endoscopy and biopsies for persistent symptoms showed ulceration, granulation tissue, fibrosis and neutrophilic inflammation; special stains and viral immunohistochemistry were negative. Endoscopic dilatation was undertaken.	Infection, malignancy and classic Crohn’s features were not demonstrated.
2024–2025	Serial CT abdomen/pelvis studies demonstrated multifocal small-bowel strictures with proximal dilatation and matted loops.	Supported a diffuse stricturing enteropathy rather than a single fixed anastomotic lesion.
July 2025	Exploratory laparotomy with adhesiolysis, strictureplasty and jejunal resection for multifocal small-bowel strictures.	Relieved obstruction and provided tissue contributing to the diagnosis of CMUSE as a diagnosis of exclusion.
August 2025	Recurrent vomiting and partial obstruction; CT showed marked gastric distension with fluid-filled dilated small-bowel loops. Gastroenterology and colorectal MDT review revisited Crohn’s disease versus CMUSE/other causes and considered bowel-preserving strategies.	Nasogastric decompression, nasojejunal feeding and nutritional optimization were instituted.
September 2025	Wound breakdown with hypodense material tracking along the laparotomy wound and evolving colocutaneous fistula.	Managed conservatively with drainage/collection control, local wound care and nutritional support because of high operative risk.
14 October 2025	Emergency wound washout, excision of granulation tissue and drainage of an adjacent abscess.	Source control was achieved and antibiotics continued.
28 October 2025	Interventional radiology revised a superficially positioned 14 Fr fistula drain.	Drainage strategy was adjusted as part of ongoing nonoperative management.
Ongoing follow-up	Regular MDT input from surgery, gastroenterology, dietetics, infectious diseases and radiology, with avoidance of radical resection where possible.	Long-term focus remained symptom control, nutritional support, infection prevention and judicious immunosuppression.

## Data Availability

No new data were created or analyzed in this study. Data sharing is not applicable to this article.
